# EXACT-Net: Framework for EHR-Guided Lung Tumor Auto-Segmentation for Non-Small Cell Lung Cancer Radiotherapy

**DOI:** 10.3390/cancers16234097

**Published:** 2024-12-06

**Authors:** Hamed Hooshangnejad, Gaofeng Huang, Katelyn Kelly, Xue Feng, Yi Luo, Rui Zhang, Ziyue Xu, Quan Chen, Kai Ding

**Affiliations:** 1 Department of Biomedical Engineering, Johns Hopkins University, Baltimore, MD 21218, USA; 2 Department of Radiation Oncology and Molecular Radiation Sciences, Johns Hopkins University, Baltimore, MD 21287, USA; 3 Carina Medical, Lexington, KY 40509, USA; 4 Department of Surgery, University of Minnesota, Minneapolis, MN 55455, USA; 5 NVIDIA Corp., AI Infrastructure, Santa Clara, CA 95050, USA; 6 Department of Radiation Oncology, Mayo Clinic, Phoenix, AZ 85054, USA

**Keywords:** multi-modal AI, lung tumor auto-segmentation, large language models, lung cancer radiotherapy

## Abstract

Background/Objectives: Lung cancer is a devastating disease with the highest mortality rate among cancer types. Over 60% of non-small cell lung cancer (NSCLC) patients, accounting for 87% of lung cancer diagnoses, require radiation therapy. Rapid treatment initiation significantly increases the patient’s survival rate and reduces the mortality rate. Accurate tumor segmentation is a critical step in diagnosing and treating NSCLC. Manual segmentation is time- and labor-consuming and causes delays in treatment initiation. Although many lung nodule detection methods, including deep learning-based models, have been proposed. Most of these methods still have a long-standing problem of high false positives (FPs). Methods: Here, we developed an electronic health record (EHR)-guided lung tumor auto-segmentation called EXACT-Net (EHR-enhanced eXACtitude in Tumor segmentation), where the extracted information from EHRs using a pre-trained large language model (LLM) was used to remove the FPs and keep the TP nodules only. Results: The auto-segmentation model was trained on NSCLC patients’ computed tomography (CT), and the pre-trained LLM was used with the zero-shot learning approach. Our approach resulted in a 250% boost in successful nodule detection using the data from ten NSCLC patients treated in our institution. Conclusions: We demonstrated that combining vision-language information in EXACT-Net multi-modal AI framework greatly enhances the performance of vision only models, paving the road to multimodal AI framework for medical image processing.

## Simple Summary

In recent years, large language models have shown great potential to enhance traditional medical image processing by incorporating multimodality information into decision-making. Conventional artificial intelligence systems solely rely on images to make predictions or decisions. However, information from medical reports can provide invaluable information for the system to curate its decision. Here we are presenting a multimodality language-vision model and framework for accurate segmentation of medical images.

## 1. Introduction

Lung cancer is the deadliest cancer type, with more than 238,000 new diagnoses and 124,000 (more than 50% of diagnoses) each year in the US [1]. Radiation therapy (RT) is the common and preferred treatment modality for medically inoperable non-small cell lung cancer (NSCLC) [2], accounting for 87% of lung cancer cases [3]. Over 60% of NSCLC diagnoses, more than 142,000 patients, require RT at least once over the course of their disease [4,5]. However, the current RT workflow is time-consuming and comprises numerous steps, resulting in a considerably long time to treatment initiation (TTI).

Previous studies have reported that lung cancer mortality rate increases with prolonged TTI [6,7,8,9,10,11], by increasing the chance of new lymph node involvement, site of disease, and also restaging [12]. Demonstrating that reducing the TTI is critical for patient survival and treatment outcomes.

**Figure 1:**
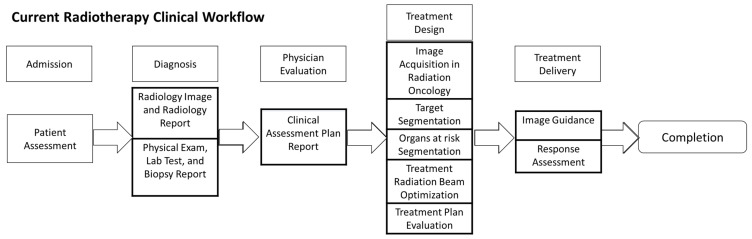
The current radiotherapy workflow consists of multiple steps, including the target and organs at risk segmentation.

Tumor segmentation is a vital step in the diagnosis and treatment workflow. Figure 1 shows the current radiotherapy workflow, which starts with patient registration and assessment. The patient is then sent for radiology diagnostic image acquisition and pathology tests. The radiologist and pathologist provide their initial diagnosis based on the test as a report for the physician’s evaluation. Based on the physician’s clinical evaluation and radiology and pathology reports. A clinical assessment plan report is generated that not only includes the diagnostic reports but also the concluding diagnosis. To design the treatment, a new patient’s CT simulation scan is acquired, and then the pivotal step of target and organs at risk segmentation is carried out, which is critical for radiotherapy plan optimization and effectiveness of treatment. Treatment is delivered with image guidance, and the treatment response is assessed for potential amendment. If no further treatment is required, the treatment will be concluded.

The main imaging modality used for diagnosis and RT treatment is computed tomography (CT). Since CT scans are very high quality with thin slices, they are large volumetric scans with millions of voxels, making the diagnosis and tumor segmentation challenging and time-consuming even for radiologists [13]. Given that manual segmentation is labor-intensive and considerably time-consuming, an automated lung cancer nodule segmentation method is extremely desirable.

Although there have been numerous efforts for automatic lung nodule segmentation, a major and long-lasting issue with the automatic methods is their high false positive (FP) rate. Pulmonary blood vessels, lung borders, and noise from CT scanners may result in high false-positive rates of detected nodules [14]. As a result, most algorithms are comprised of nodule detection and FP reduction systems [13,14,15,16] or are dealt with by manual cropping before nodule segmentation [17]. Different methods such as classic machine learning, feature extraction [18], and deep learning (DL) [19] algorithms have been used for FP reduction, but there are still challenges with these methods.

Meanwhile, information pinpointing the location of the lung tumor is already available in the patient’s electronic health record (EHR), such as clinical decisions outside the traditional simulation CT (such as reports from diagnostic radiology scans and pathology reports). Still, little attention has been paid to parsing and incorporating this data into target delineation. These reports have been meticulously written and contain valuable information regarding the location, shape, and size of the tumor.

Recently, large language models (LLMs) have shown remarkable performance in a variety of natural language processing (NLP) tasks, such as summarization, questioning, and answering [20]. Using neural networks with several billion parameters and self-surprised learning on a large corpus of unlabeled text data. LLMs can very efficiently parse information and generate human-like responses to prompts. OpenAI GPT3 and 4, Google Bard [21], and Meta LLAMA are only a few examples of state-of-the-art LLMs.

ChatGPT is developed from InstructGPT [22] by fine-tuning the dialog interface. While InstructGPT is designed for processing prompts to provide responses to pre-defined tasks and instructions, ChatGPT is designed to engage in conversion and create more naturalistic responses [22]. ChatGPT is trained on more than 300 billion words and has more than 100 trillion parameters [23], showing remarkable capability in generating and collaborating with users for creative and technical downstream tasks. Recent findings have shown that with in-context zero-shot and few-shot leanings, LLMs can adapt to many novel downstream tasks [24,25].

“Prompt Engineering” is an important aspect of zero-shot and few-shot learning, which is best defined as providing effective contextual cues to help the LLMs with the given task. More recently, LLMs have also been used to produce clinical reports [26,27] or, more focused, generate domain-specific text, such as radiation oncology treatment regimens [28].

**Figure 2:**
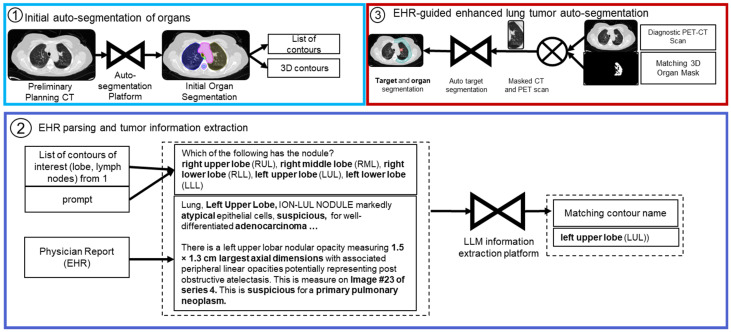
Overview of an EHR-guided automated target segmentation system. The auto-contouring platform contours the initial structures on the diagnostic CT scan. Due to the advantages of PET scans for improved target segmentation, it will be used as the second primary imaging modality for the target segmentation platform. The NLP-based algorithm will extract critical information regarding the location and shape of the tumor.

In this work, we demonstrate the feasibility of an EHR-guided lung tumor auto-segmentation method named EXACT-Net, or EHR-enhanced eXACtitude in Tumor segmentation (Figure 2). To deal with the critical issue of FPs in lung nodule segmentation, LLM-parsed patient-specific EHR is used to aid the tumor auto-segmentation method to eliminate the FP nodules from the result. This is because the main tumor to be treated can be one of the detected nodules, and not all lung nodules are cancerous tumors. Thus, without relying on direct user input, FPs were significantly reduced, and automatic target segmentation was improved. Specifically, the LLM-extracted tumor location served as an input bounding area for the CT images to guide the segmentation network.

In the following, we review the related works for lung tumor segmentation and attempts that have been made to improve the lung segmentation nodule detection frameworks (Section 2). In Section 3, we talk about the method used in this work to create a multimodality long tumor segmentation method and how its effectiveness is validated. Section 4 and Section 5 are dedicated to the results and discussion regarding the strengths and weaknesses of our method and future work. Finally, in Section 6, we provide a summary of this work and conclusions.

## 2. Related Work

Lung cancer is a devastating disease, and tumor segmentation is a critical part of radiotherapy treatment practice, as initiating the treatment depends on the tumor and organs at risk segmentation. Thus, lung tumor segmentation has been of much interest to many researchers over the years. The first attempts at lung tumor segmentation were based on image characteristics such as shape, intensity, and texture. These methods were computationally expensive and hard to generalize to difficult cases and were mainly based on traditional image processing methods such as intensity and adaptive thresholding, image registration, and region-growing [29,30].

CT scan, amongst all the other imaging modalities, is the standard of care for cancer therapy such as radiation therapy [31,32,33]. The diagnostic CT scan, which is acquired for tumor delineation, has a field of view that is bound to the lungs to make lung parenchyma easily distinguishable. However, even with this field of view, tumors only account for a small number of voxels compared to the entire CT, making lung tumor segmentation very challenging [34,35].

Convolutional neural networks (CNNs) have been used in numerous medical image processing fields, such as segmentation, classification, detection, and reconstruction, and have achieved great success. CNNs have been applied to lung CT scans for lung pathology segmentation [36], lung volume segmentation [37], and lung region classification [36,38]. In the case of entire lung volume segmentation, there are two common approaches: (i) 2D segmentation, in which each CT slice is segmented independently. In this case, the CNN architectures, such as UNet or VGG-16, are used to segment each slice of CT in a 2D manner [37,39,40]. Generative adversarial networks (GAN) have also been used for segmentation, where the GAN model is used to encode features of the CT slice and then the encoder-decoder is used to segment lung volume [41]; (ii) 3D segmentation, in which the CT scan is treated as 3D volume and is segmented for lung in 3D. Previously, V-net and more improved networks such as RU-Net and R2U-Net have been used for volumetric segmentation [42,43,44].

In the case of lung tumor segmentation, the common practice is a 2-step framework in which a delineation model first segments all suspicious tumors, and an FP reduction model reduces the FPs. For instance, Xie H. et al. [45] used a 2D Faster R-CNN model with deconvolutional layers to magnify the feature maps to detect all the candidate nodules from slices of CT scan. Then, they trained a classifier to reduce FPs. Another work used a different architecture, ResNet [46], to segment tumors for NSCLC cases. For 3D tumor segmentation, Kopelowits E. et al. [47] used MaskRCNN to detect 3D nodules on CT scans, and Kamal U. et al. [48] used a recurrent 3D-DenseUNet for lung tumor segmentation. More recently, Le V. and Saut O. [13] used a variant of the UNet model, RRc-UNet 3D, which is augmented with residual recurrent block, for 3D segmentation of NSCLC tumors.

## 3. Materials and Methods

Here we have developed a 2-step framework for an EHR-guided auto-target segmentation, where an LLM model extracts tumor location, tumor size, and lymph node involvement from clinical reports (Figure 2), and the parsed data from LLM, such as tumor location and size, be used to refine the result and a robust target delineation.

### 3.1. LLM Tumor Phenotype Extraction

To demonstrate the feasibility of EHR-guided tumor segmentation, we used the GPT 3.5 Turbo LLM through the OpenAI API. We used an in-house Python interface to the API to read the EHR text files and feed them to the model. To access the API, an API key is required that can be obtained from the OpenAI official website (https://platform.openai.com/docs/model-index-for-researchers (accessed on 25 October 2023)).

An important step in utilizing a Large Language Model (LLM) effectively is setting up the appropriate parameters before submitting the prompts. One of the most crucial parameters is the “Temperature”, which significantly influences the model’s output. The temperature parameter controls the randomness of the predictions made by the model. A higher temperature value (closer to 1) results in more diverse and creative responses, as the model takes more risks in generating outputs. Conversely, a lower temperature value (closer to 0) makes the model’s responses more deterministic and focused, as it tends to choose the most probable next word in a sequence [49].

In our specific use case, achieving maximum certainty in the model’s responses is highly desirable. This is because we prioritize accuracy and consistency over creativity. To ensure this, we set the temperature parameter to zero. By doing so, we instruct the model to produce the most predictable and reliable outputs, minimizing the chances of unexpected or varied responses. This approach is particularly beneficial in scenarios where precise and unambiguous information is required, such as technical documentation, legal texts, or any context where clarity and correctness are paramount. To demonstrate the feasibility, the pathology lab and radiology image reports and CT images of 10 cases of lung cancer patients treated with RT at Johns Hopkins Hospital were used for this study. As, at the moment, we did not have the resources to deploy the local model, we manually de-identified all the patient data, removed the names and locations, and altered the dates of the procedures and visits.

We implemented our novel strategy using the zero-shot learning approach, meaning that we have not fine-tuned the model specifically for cancer reports. Zero-shot learning [50] allows the model to make predictions on new, unseen data without any prior training on that specific type of data. This approach leverages the extensive pre-training of the model on a diverse range of texts, enabling it to generalize well to new tasks.

Given that the model has not been fine-tuned for our specific use case, an important step is “prompt engineering”. Prompt engineering involves the careful design and crafting of prompts that interact with ChatGPT to guide the pre-trained GPT-3.5 model in extracting the relevant information. A prompt is essentially an instruction set that regulates the LLM’s capabilities and significantly affects the generated outputs. Effective prompt engineering can enhance the model’s performance by providing clear, concise, and contextually appropriate instructions [51].

Prompts serve multiple functions. They can filter information by specifying the type of data or response required, ensuring that the outputs are relevant and useful. Additionally, prompts can create new interaction paradigms, such as directing the LLM to search for and synthesize information that it has not explicitly seen before. This capability is particularly valuable in fields such as medical research, where the ability to generate accurate and insightful responses from a broad knowledge base can aid in the analysis and interpretation of complex data [52].

By leveraging prompt engineering, we can maximize the utility of the pre-trained model, ensuring that it provides precise and relevant information even without fine-tuning. This approach not only saves time and resources but also demonstrates the versatility and robustness of the LLM in handling diverse and specialized tasks. Here, we also used prompts as a tool for LLM self-adaptation to new tasks. For instance, we can extract the tumor location by using prompts such as “Find the current lung lobe that the determinate tumor/carcinoma/malignancy is involved in this report:” or ”Based on the report, where is the tumor or carcinoma located?” followed by possible options of right upper lobe (RUL), right middle lobe (RML), right lower lobe (RLL), left upper lobe (LUL), left lower lobe (LLL). Currently, in the medical domain, automatically learned prompts are less common as they are often not human-readable [53]. As interpretability is essential, we manually engineered prompts with trial and error.

### 3.2. Tumor Auto-Segmentation Algorithm Design and Training

**Figure 3:**
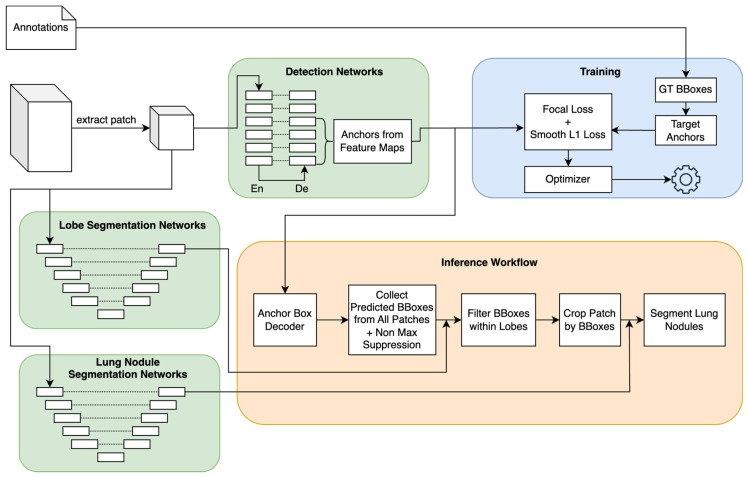
Detailed tumor auto-segmentation model architecture.

To automatically detect and segment the lung nodules, we developed an automated deep-learning model. We chose to employ the UNet3D architecture for our 3D segmentation networks (Figure 3). This selection was based on the architecture’s well-established effectiveness in segmenting 3D medical images [54,55]. 2D object detection in computer vision has significantly advanced and matured. When it comes to 3D object detection in medical images, recent efforts have drawn inspiration from the progress in 2D single-stage detectors, particularly RetinaNet [56], and leveraged the successful 3D segmentation models, UNet3D, to construct a 3D detection framework termed Retina-UNet3D.

RetinaNet stands out due to its feature pyramid network (FPN), which efficiently handles object detection across various scales. This FPN-based approach serves as the fundamental building block for our 3D object detection framework, providing a strong foundation for our work in the medical image domain. In the context of 3D segmentation, the UNet3D encoder-decoder approach has proven highly effective. Leveraging this success, Retina-UNet3D adopts the UNet3D encoders and establishes connections between the encoder outputs and the detection decoder heads, as the UNet architecture also shares a similar pyramid network design.

This design choice aligns with the principles of both FPN and UNet, allowing us to leverage the benefits of feature pyramid structures for our 3D detection framework while building upon the successful foundation of UNet3D in segmentation tasks.

### 3.3. Loss Functions

In our segmentation tasks, we used a dual loss strategy:

Categorical Cross-Entropy Loss: This loss is employed for pixel-level classification, ensuring precise object boundary delineation and class-specific region identification.

(1) $$L\left(p,q\right)=-\sum_{i}y_{i}\log\left(p_{i}\right)$$

Dice Loss: Dice loss is used for image-level Intersection over Union (IoU) measurement, assessing overall segmentation quality.

(2) $$L\left(y,p\right)=1-\frac{2\sum_{i}y_{i}p_{i}}{\sum_{i}y_{i}^{2}+\sum_{i}p_{i}^{2}}$$

These two losses are in the same order of magnitude, so we simply added them as the final dual loss. This approach stroked a balance between pixel-wise accuracy and image-level fidelity, enhancing segmentation performance across both detailed object recognition and holistic quality assessment.

In our detection tasks, we used smooth L1 loss [57] for box regression heads and focal loss [56] for box classification heads. Hereby, we used δ=1. MAE is the mean absolute error between predicted bounding boxes and ground-truth bounding boxes.

(3) $$SmoothL1=\left\{\begin{array}{c} \frac{0.5{\left(MAE\right)}^{2}}{\delta }\;\;\;\;\;\mathrm{if}\;\mathrm{MAE}<\delta \\ \mathrm{MAE}-0.5\delta \;\;\;\;\;\;\;\mathrm{otherwise} \end{array}\right.\;and\;MAE\left(x,y\right)=\frac{\sum_{1}^{N}\left|x_{i}-y_{i}\right|}{N}$$

(4) $$FL\left(p,y\right)=\left\{\begin{array}{c} -\alpha {\left(1-p\right)}^{\gamma }\log\left(p\right)\;\;\;\;\;\;\;\;\mathrm{if}\;y=1\\ -\left(1-\alpha \right)p^{\gamma }\log\left(1-p\right)\;if\;y=0 \end{array}\right.$$

### 3.4. Dataset and Data Pre-Processing

We used the publicly available Lung Image Database Consortium imaging collection (LIDC-IDRI) from TCIA [58] as it contains high-quality annotations for all nodules ≥3 mm with consensus from experienced radiologists. With the same LIDC-IDRI dataset, [59] annotated 50 CT scans on lobe segmentation.

We standardized the voxel spacings of all CT datasets to 1 mm × 1 mm × 1 mm. In addition, we applied intensity clipping, constraining the Hounsfield Units (HU) within the range of −1000 HU to 600 HU. This adjustment was made to prevent the model from overly emphasizing irrelevant information.

Furthermore, in preparation for training our detection model, data containing segmentation contours were transformed into bounding boxes. This conversion facilitated the training process and enabled effective detection.

### 3.5. Data Augmentation

For our segmentation task, we employed a comprehensive data augmentation approach, which included 3D random rotations within a range of −30 to 30 degrees along each axis, random scaling between 70% and 140%, random cropping, random flipping along each axis, random Gaussian noise, random Gaussian blur, and random variations in brightness, contrast, and gamma.

For our detection task, we used the same augmentation methods as segmentation but removed rotation and flipping approaches. This combined augmentation strategy was instrumental in enhancing the robustness and generalization of our models.

### 3.6. Training and Evaluation Methods

Our physical device was equipped with a Nvidia RTX 4080 with 16 GB of memory. To address the considerable memory demands associated with 3D medical images, we adopted a sliding patch method, enabling us to effectively train models on large-sized images [54,60].

For the lung nodule detection model, training was conducted over 500,000 iterations, employing patch sizes of 96 × 96 × 96 (equivalent to 96 mm × 96 mm × 96 mm) with a batch size of 1. In the case of the lung nodule segmentation model, it underwent training for 300,000 iterations, with patch sizes set at 64 × 64 × 64 (64 mm × 64 mm × 64 mm) and a batch size of 1.

Similarly, the lobe segmentation model was trained for 300,000 iterations, with patch sizes configured as 96 × 192 × 192 (equivalent to 288 mm × 192 mm × 192 mm, with 3× down-sampling along the axial axis) and a batch size of 1. These training settings were tailored to accommodate the hardware limitations while ensuring efficient and effective model training.

Our inference workflow was structured into two connecting stages: detection and segmentation. To ensure consistency, we began with the same preprocessing steps, which involved voxel standardization and intensity clipping for the input data.

In the first stage, we employed the lung nodule detection model to generate a list of bounding box candidates for potential nodules. Subsequently, we applied the lobe segmentation model to obtain lobe masks and assigned lobe location information to each bounding box. Any candidate bounding box lacking contact with a lobe was discarded from further consideration.

In the second stage, we cropped 64 × 64 × 64 patches for each remaining candidate. These patches were then processed using the lung nodule segmentation model to delineate the contours of the nodules. This two-stage approach allows us to first identify potential nodules and then precisely segment their contours.

We assessed the quality of segmentation results using the Dice Coefficient Score (DSC), which provides a measure of the overlap between predicted and ground-truth segmentations.

(5) $$DSC=\frac{\left|x\cap y\right|}{\left|x\right|+|y|}$$

For our detection results, we employed the mean average precision (mAP) metric with two intersection over union (IoU) thresholds at 0.5 and 0.7 for 3D objects. An IoU of 0.5 signifies a good match for 3D small objects, and an IoU of 0.7 represents an excellent match. These IoU thresholds allow us to precisely assess the performance of our detection model across varying levels of accuracy and provide a comprehensive evaluation of its object detection capabilities.

(6) $$mAP=\frac{1}{N}\sum_{c=1}^{N}\frac{TP\left(c\right)}{TP\left(c\right)+FP(c)}$$

As we only had one class of detection object, mAP is equivalent to AP.

### 3.7. Experiment Design

We verified our approach and demonstrated the effect of EHR-extracted information on the tumor segmentation model performance by conducting a comparison study. We used data from 10 test patients from the Johns Hopkins Hospital database, unseen by both the LLM and tumor auto-segmentation model. Once the nodules were detected and classified using the entire chest CT scan without any EHR guidance. Then the entire body was automatically segmented using the anonymized patients’ diagnostic CT scan for all organs, including lung lobes and lymph nodes. Next, auto-lung nodule detection and classification software detected and classified all supposedly malignant nodules.

Next, using the EHR-extracted information, we only presented the auto-segmentation model with the confirmed location of the tumor according to the clinical reports. For instance, if reports mention that the tumor is in the “left upper lobe (LUL)”, the LUL mask was used to mask all other structures, and only the LUL was presented to the segmentation model for nodule detection.

## 4. Results

### 4.1. Tumor Auto-Segmentation Performance

The evaluation of segmentation results using the Dice Coefficient Score (DSC) revealed that lobe segmentation closely approximated the manual annotations made by radiologists. This high level of performance in lobe segmentation significantly reinforced the viability of our experiments, which focused on tumor identification based on lobe-specific information obtained from radiology reports.

In comparison to lobes and lungs, lung nodules are notably smaller in size. It is important to note that smaller regions tend to yield lower DSC scores (Table 1), given the inherent challenges in segmenting small structures accurately. Therefore, achieving a DSC score of 0.67 for lung nodule segmentation can be considered a favorable outcome (Table 2).

The results were measured in the validation subset of LIDC-IDRI with 201 CTs and 399 solid nodules. Our lung nodule detection model has demonstrated strong performance in identifying solid nodules. However, it is important to note that, in this process, some false positives may arise, and there may be challenges in detecting ground glass nodules.

### 4.2. LLM Prompt Design

To do so, first, as an “assistant prompt”, we gave a list of lung lobes as options to direct pre-trained LLM to find the lobe with the tumor. Secondly, we used the following as the “user prompt”: “Find the current lung lobe that the determinate tumor/carcinoma/malignancy is involved in this report:”.

There are three keywords in this prompt to effectively guide pre-trained LLM, (i) current, as the patient may have a history of several lung tumor treatments, it is important to specify the currently under treatment tumor; (ii) determinate, the patient reports may include some indeterminate nodules, which we are not interested in for current treatment; (iii) tumor/carcinoma/malignancy, we used the common words used in the report to refer to tumors in reports so that we do not miss any information regarding tumor location.

Furthermore, to find the malignant lymph nodes, we specifically used the pathology report with the following “user prompt”: “Find out what lymph station/node is malignant in this report:”.

With the above-mentioned prompt, we could find the right tumor location with 100% accuracy, and it is important to consider that this is based on zero-shot learning, which means no fine-tuning has been performed on the model. Again, we could find the involved lymph nodes with 100% accuracy from the reports.

### 4.3. EHR-Guided Tumor Auto-Segmentation Experiment

Table 3 summarizes the results for the ten test patients. As seen, when the tumor segmentation algorithm is used without any EHR information, in only 20% of patients the result matches the ground truth, and similar to previous models, there is a false positive issue with initial nodule detection. On the other hand, when EHR-extracted information is used to guide tumor detection, for 70% of the patients, the result matches the ground truth. Thus, in other words, using medical report information resulted in a huge increase in the number of successful auto-tumor segmentation cases and a 250% boost in performance, demonstrating the power of our approach in reducing the FPs.

**Figure 4:**
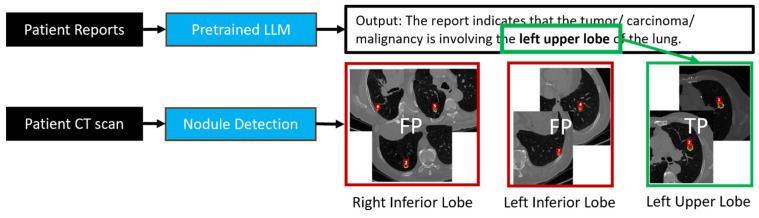
Showing an example case of EHR-guided tumor auto-segmentation where the extracted information regarding the confirmed tumor is used to remove FPs. The nodule detection algorithm detected and classified seven nodules (red circles) as malignant for this sample case (Case ID 4). Out of the seven nodules, three were found in the right inferior lobe (RIL, left red box), two were found in the left inferior lobe (LIL, middle red box), and two in the left upper lobe (LUL, right green box). According to the EHR-extracted information, however, the confirmed tumors were in LUL. Using this information, the result is automatically refined, and the FPs are removed.

Figure 4 shows a sample case (Case ID 4), where the nodule detection algorithm detected and classified seven nodules as malignant. Out of the seven nodules, three were found in the right inferior lobe (RIL), two were found in the left inferior lobe (LIL), and two in the left upper lobe (LUL). According to the EHR-extracted information, however, the confirmed tumors were in LUL. Using this information, the result is automatically refined, and the FPs are removed.

## 5. Discussion

In this study, we presented a novel EHR-guided tumor auto-segmentation method. We showed that incorporating the EHR information into segmentation, as prior knowledge of confirmed tumor location, is very effective in reducing the FPs. We evaluated the method using the data from ten test cases of actual patients from our institution’s database.

**Figure 5:**
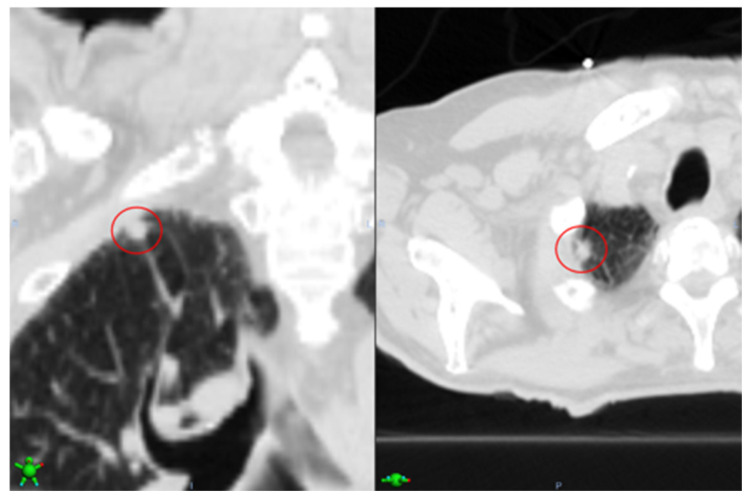
Case 6 where the nodule is very close to the chest wall, making the nodule detection very challenging. Left showing the coronal view, and right axial view, with tumor marked with red circle.

We are aware that our study may have a few limitations. First, as seen in Table 3, we see two cases (6 and 10) with false negatives (FN), which is highly undesirable. Upon further analysis, we realized that for case 6, the reason that the segmentation model did not detect the nodule is that, (i) as seen in Figure 5, the nodule is very close to the lung wall, making the nodule detection very challenging, which has also been reported by previous works [13]; (ii) currently, our model is designed as a standalone nodule detector, thus it is restrictive to detect fewer FPs, which resulted in some True Positives being filtered out. With the EHR-extracted information and lowering the classifier threshold, the issue is resolved.

**Figure 6:**
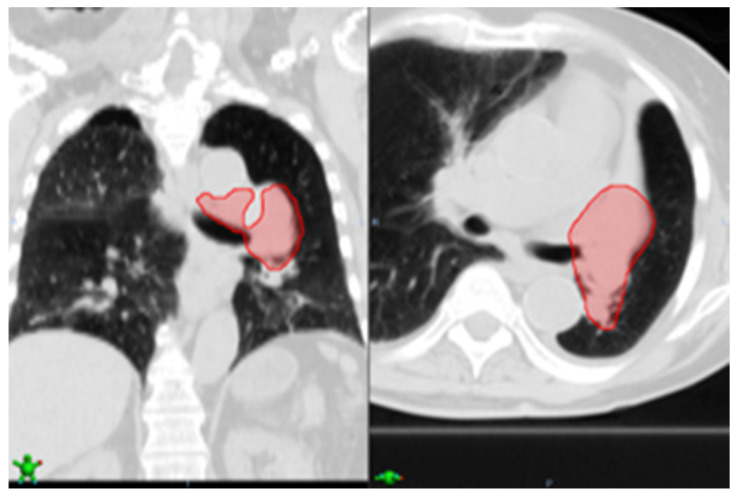
Case 10, our approach averted a false positive in the right upper lobe; however, due to highly advanced stage 4 cancer, the nodule detection failed to detect the advanced tumor shown in the red shade (left coronal view, right axial view).

As for case 10 (Figure 6), the nodule detection method found an FP nodule in the right upper lobe. According to the EHR report, the tumor was in the left upper lobe, thus the FP was averted. However, case 10, particularly, is highly advanced stage 4 and thus very challenging. As seen in Figure 6, the tumor spread a lot and involved a large area of lymph nodes. Because currently our model is not trained on these challenging cases, it failed to detect the tumor.

## 6. Conclusions

In this study, we introduced an innovative method to improve the performance of nodule detection by integrating information extracted from Electronic Health Records (EHR). Our approach, named EXACT-Net, leverages a pre-trained Large Language Model (LLM) to accurately identify and extract clinically confirmed tumor locations from EHR data. This extracted information is then utilized to filter out false positives (FPs) in the nodule detection process.

We employed a zero-shot learning strategy, utilizing prompt engineering to effectively extract the necessary information without requiring additional training data. This approach allows EXACT-Net to significantly reduce the occurrence of false positives, addressing a persistent challenge faced by most existing nodule detection algorithms.

By incorporating EHR-extracted information, EXACT-Net enhances the accuracy and reliability of nodule detection, offering a promising solution to a long-standing problem in the field. This method not only improves diagnostic precision but also has the potential to streamline clinical workflows and improve patient outcomes.

## Figures and Tables

**Table 1:** Dice score of the auto segmentation method.

Lung Region	DSC
RUL	0.94
RML	0.83
RLL	0.96
LUL	0.98
LLL	0.97
Lungs Overall	0.97
Lung Nodule	0.67

**Table 2:** Lung nodule detection performance.

Region of Interest	AP@IoU = 0.5	AP@IoU = 0.7
Lung Nodule	65.63	59.15

**Table 3:** The EHR-guided tumor segmentation experiment result.

Case ID	Ground Truth	Detected Nodules	Removed Nodules	Matching Ground Truth
1	1	2 (FP)	1	Yes
2	1	1	0	Yes
3	1	1	0	Yes
4	2	7 (FP)	5	Yes
5	2	4 (FP)	2	Yes
6	1	0 (FN)	0	No
7	2	5 (FP)	3	Yes
8	1	4 (FP)	2	No (FP)
9	1	3 (FP)	2	Yes
10	1	1 (FP)	1	No

## Data Availability

Raw data supporting the conclusions of this article will be made available by the authors upon request.
